# Biocompatible ZnS:Mn quantum dots for reactive oxygen generation and detection in aqueous media

**DOI:** 10.1007/s11051-015-3269-x

**Published:** 2015-11-30

**Authors:** Daysi Diaz-Diestra, Juan Beltran-Huarac, Dina P. Bracho-Rincon, José A. González-Feliciano, Carlos I. González, Brad R. Weiner, Gerardo Morell

**Affiliations:** Molecular Sciences Research Center, University of Puerto Rico, San Juan, PR 00926 USA; Department of Chemistry, University of Puerto Rico - Río Piedras, San Juan, PR 00936 USA; Department of Physics, University of Puerto Rico - Río Piedras, San Juan, PR 00936 USA; Department of Biology, University of Puerto Rico - Río Piedras, San Juan, PR 00931 USA; Department of Biochemistry, School of Medicine, University of Puerto Rico – Medical Sciences, San Juan, PR 00936 USA

**Keywords:** ZnS:Mn, Biosensing, Photodynamic therapy, Quantum dots, Singlet oxygen, Tyrosinase

## Abstract

**Abstract:**

We report here the versatility of Mn-doped ZnS quantum dots (ZnS:Mn QDs) synthesized in aqueous medium for generating reactive oxygen species and for detecting cells. Our experiments provide evidence leading to the elimination of Cd-based cores in CdSe/ZnS systems by substitution of Mn-doped ZnS. Advanced electron microscopy, X-ray diffraction, and optical spectroscopy were applied to elucidate the formation, morphology, and dispersion of the products. We study for the first time the ability of ZnS:Mn QDs to act as immobilizing agents for Tyrosinase (Tyr) enzyme. It was found that ZnS:Mn QDs show no deactivation of Tyr enzyme, which efficiently catalyzed the hydrogen peroxide (H_2_O_2_) oxidation and its eventual reduction (−0.063 V vs. Ag/AgCl) on the biosensor surface. The biosensor showed a linear response in the range of 12 μmol/L–0.1 mmol/L at low operation potential. Our observations are explained in terms of a catalase-cycled kinetic mechanism based on the binding of H_2_O_2_ to the axial position of one of the active copper sites of the *oxy*-Tyr during the catalase cycle to produce *deoxy*-Tyr. A singlet oxygen quantum yield of 0.62 in buffer and 0.54 in water was found when ZnS:Mn QDs were employed as a photosensitizer in the presence of a chemical scavenger and a standard dye. These results are consistent with a chemical trapping energy transfer mechanism. Our results also indicate that ZnS:Mn QDs are well tolerated by HeLa Cells reaching cell viabilities as high as 88 % at 300 µg/mL of QDs for 24 h of incubation. The ability of ZnS:Mn QDs as luminescent nanoprobes for bioimaging is also discussed.

**Graphical Abstract:**

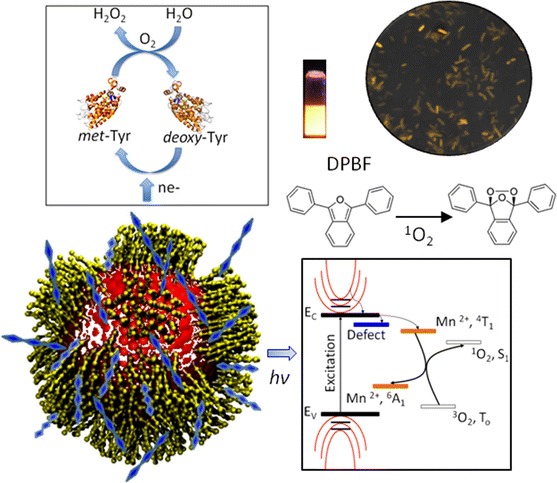

## Introduction

Quantum dots (QDs) are recently being envisioned as potential photosensitizers (PS) for photodynamic therapy (PDT) to treat cancer (Thakor and Gambhir 2013; Allison and Moghissi 2013; Chen et al. 2013), due to their flexible bioconjugation, biological targeting efficiency, high quantum yield (QY), broad absorption with relatively narrow, symmetric emission, and long-term stability (Sotelo-Gonzalez et al. 2013; Zhang et al. 2011; Zhuang et al. 2003; Shen et al. 2012). The availability of QDs whose optical properties can be tuned by size, composition, and doping has brought forth new pathways to produce reactive oxygen species (ROS), in particular singlet oxygen (^1^O_2_), by photochemical methods. As a proof of concept, many QDs have been tested, but a strong focus has been placed on CdSe and CdTe QDs. For instance, CdSe QDs can be used to sensitize molecular oxygen through a triplet energy transfer mechanism. They can produce a ^1^O_2_ QY of ~5 % in toluene (cf. 43 % in Pc4 PS used in clinical trials), and their optical properties can be tuned to treat both shallow- and deep-seated tumors (Samia et al. 2003). Shi et al. working with CdTe QDs electrostatically bound to *meso*-tetra(4-sulfonatophenyl)porphine dihydrochloride (PS) obtained a ^1^O_2_ QY of ca. 43 % (being ≥40 % reported for traditional PSs) (Shi et al. 2006). Nonetheless, they did not fully quantify their results. Still, the cytotoxicity and solubility in water of Cd-based QDs (despite extended efforts to modify their surface) remain a significant concern. In this regard, Hsiech et al. overcoated CdSe cores with ZnS, being then attached to a cyclometalated Ir complex-type sensitizer (Hsieh et al. 2006). Although the ^1^O_2_ QY in benzene was improved, it was the Ir complex that was directly excited instead of the QDs. It has been reported that ZnS shells greatly reduce the toxic effects of Cd-based QDs in live cells and provide an improved ^1^O_2_ QY (as high as 31 %) (Tsay et al. 2007; Kirchner et al. 2007). Nevertheless, they are still tested in non-aqueous solutions due mainly to the surface chemistry of the core material. We have thus investigated the possibility to omit the use of Cd-based cores and substitute their optical contribution by Mn doping in ZnS (Bhargava and Gallaguer 1994). Recently, Zhou et al. reported on the sensitizing effect of cysteine-capped Mn-doped ZnS (ZnS:Mn)/Si QDs via the ^1^O_2_ quenching method (Zhou et al. 2011), but the findings of this exploratory work need further confirmation. The omission of the Si coating and evaluation of the quantification process of ^1^O_2_ using ZnS:Mn QDs via photooxidation are still a challenge.

Due to the biocompatibility and high QY (compared to Cd-based QDs) of ZnS:Mn QDs, they are also employed as nanoprobes in order to better understand their main “nano-bio” interaction with cells and bacteria. It has been reported that ZnS:Mn QDs offer improved ability to image single cancer cells and colonies without causing any effect on their metabolic activity and morphology (Mathew et al. 2010; Manzoor et al. 2009). Correlated reports have shown that ZnS:Mn capped with different ligands possesses a great capability to image *Staphylococcus aureus*, exceptional potential to be used for a rapid screening of metal-accumulating *Lysinibacillus fusiformis* cells, and intrinsic inhibiting effect on the growth of *Escherichia coli* (Sharma et al. 2010; Sajimol et al. 2013; Kong et al. 2011; Baruah et al. 2012; Li et al. 2004). As a result, ZnS:Mn QDs can be effectively employed to detect cells, especially which are associated to the formation of biofilms. The identification of *P. aeruginosa* cells has aroused a great deal of interest to the scientific community due to their adherence to surfaces (forming biofilms) of nosocomial elements (e.g., catheters, heart valves, and prostheses), and their potential pathogenesis in patients suffering from cystic fibrosis, both constituting a critical health-related issue in hospitals (Cornelis 2008). However, no reports on labeling *P. aeruginosa* cells using biocompatible ZnS:Mn QDs have appeared in the literature to date.

ZnS:Mn QDs exhibiting highly active surface areas with an isoelectric point (IEP) of 7.2 are also attractive as innovative matrixes to immobilize enzymes with low IEP, since their interaction is mainly electrostatic. When immobilizing such large biomolecules on solid substrates, it is desirable to retain their electroactive response on the modified electrode surface, and avoid any process related to irreversible denaturalization. Tyrosinase (Tyr) is one of the most used enzymes for the modification of metal electrodes due to its intrinsic enzyme specificity and ability to catalyze the oxidation of many substrates by phenols (Jang et al. 2010). The effective immobilization of Tyr by ZnS:Mn QDs would thereby be a promising alternative given their excellent mechanical, chemical, and thermal stability over prolonged periods (Chauhan et al. 2011). The assembly of Tyr/ZnS:Mn-based amperometric biosensors to detect ROS (such as hydrogen peroxide, H_2_O_2_) may facilitate a more effective enzymatic binding, improve the properties of the bioactive layer associated with the transducer, and lead to greater efficiency in terms of sensitivity, selectivity, stability, and simplicity (Vreeke et al. 1992; Garguilo et al. 1993). Although some nanostructured immobilizing matrixes have been proposed to enhance the electron transfer rate (Jang et al. 2010; Liu et al. 2011; Zhou et al. 2010; Song et al. 2010); most of them still show low reusability and storage stability, and poor long-term stability due to the large electrochemical prosthetic groups deeply embedded into the structure of the enzyme (Chauhan et al. 2011). In order to increase the electron transfer efficiency of redox enzymes, QDs have recently been proposed due to their inherent large surface-to-volume ratio, high surface reaction activity, and strong absorption ability, which increases the binding site on the electrode surface (Chauhan et al. 2011; Çevik et al. 2012, Xia et al. 2014). Specifically, inexpensive and environmentally benign metal sulfide QDs with sizes similar to those of the working enzymes enable excellent interaction with the active centers buried deep within the protein shells. Recently, Chauhan et al. detected organophosphorus insecticides based on ZnS-immobilized rat brain acetylcholinesterase (Chauhan et al. 2011). In the presence of acetylthiocholine chloride, ZnS QDs promoted electron transfer reactions at low working potential, catalyzed electrochemical oxidation of enzymatically formed thiocholine increasing the detection sensitivity, and exhibited long-term storage stability. Nonetheless, no further reports confirming the immobilizing enzyme characteristics of ZnS QDs have appeared in the literature. The evaluation of new electron transfer paths employing more stable surface-passivated ZnS:Mn QDs is still a challenge, for instance, for the detection of H_2_O_2_ at low concentrations.

We report here on the versatility of luminescent water-soluble ZnS:Mn QDs for Tyr immobilization and multiple biological detection. The capability of ZnS:Mn QDs to produce ^1^O_2_ in the presence of 1,3-diphenylisobenzofuran (DPBS, ^1^O_2_ sensor) is presented. The ZnS:Mn QDs are also employed as nano-probes for imaging *P. aeruginosa* cells, and intended as immobilizing matrixes for Tyr enzyme by cross-linking on modified Pt electrode to detect H_2_O_2_.

## Materials and methods

### Synthesis of ZnS:Mn quantum dots

Mn-doped ZnS QDs capped with 3-mercaptopropionic acid (MPA, ≥99 %, Sigma Aldrich, USA) were prepared by an inorganic wet chemical approach reported elsewhere (Beltran-Huarac et al. 2013a, b). Briefly, 1.705 g of ZnSO_4_·H_2_O (≥99.9 %, Sigma Aldrich, USA), 0.085 g MnSO_4_·H_2_O (≥ 99 %, Sigma Aldrich, USA), and 2.61 mL MPA were dissolved into 50-mL three-neck round-bottom flask using high-purity deionized water (HPDW), resulting in a 5 at.% Mn doping. The pH of the mixed solution was adjusted to 11 using 1 M NaOH (99.99 %Sigma Aldrich, USA). After argon purging, 50 mL of 0.2 M aqueous solution of Na_2_S (Sigma Aldrich, USA) was gradually added. The mixture was stirred at room temperature with a controlled reflux system and then aged for 14 h at 50 °C. The flocculate was removed from the supernatant by ultracentrifugation, then copiously rinsed with HPDW, and dried at 60 °C overnight in order to eliminate any remaining by-product and adsorbate. The final products re-dispersed in HPDW rapidly produced a deep orange solution when exposed to UV light, which is a clear indicator of the compound formation, and were employed for further ex situ characterization. ZnS QDs were also prepared following the same recipe for comparison purposes. Peng et al. reported that this synthesis process yields an Mn doping level of 0.380 at.% when 5 at.% Mn is used in the synthesis process as measured by ICP (Peng et al. 2005).

The phase and crystalline structure of QDs were analyzed using an X-Ray Diffractometer (XRD), Model Siemens D5000. Raman spectra were collected via a Jobin–Yvon T64000 spectrometer (resolution ~1 cm^−1^) with Ar-ion laser excitation (514.5 nm), attached to an optical microscope with 80× resolution. The surface morphology, crystallite size distribution, and elemental composition were studied using a JEOL JEM-2200FS Cs-corrected high-resolution transmission electron microscope (HRTEM) operated at 200 kV. A FluoroMax-2 spectrofluorometer was employed to collect the photoluminescence (PL) spectra.

### Biosensing measurements

For the biosensor construction, the polycrystalline Pt electrode surface was mechanically polished with alumina paste (0.05 μm), washed with HPDW, and rinsed abundantly with anhydrous ethanol (≥99.5 %, Sigma Aldrich, USA). The active part of the electrode was a 4-mm-diameter disk, and the other parts were covered with isolating epoxy resin. The as-treated surface was submerged in a 10 mM solution composed of 4-aminothiophenol (ATP, 97 %, Sigma Aldrich, USA) diluted in anhydrous ethanol to form a self-assembled monolayer (SAM). Afterwards, 0.2 mg of MPA-capped ZnS:Mn QDs were linked onto SAM surface using a 2 mM solution of N,N’-dicyclohexylcarbodiimide (DCC, 60 wt% in xylenes, Sigma Aldrich, USA) in the presence of N,N-dimethylformamide (DMF, 99.8 %, Sigma Aldrich, USA). The Tyr enzyme from mushrooms (low isoelectric point, lyophilized powder, ≥1000 unit/mg solid, Sigma Aldrich, USA) was then immobilized on ZnS:Mn QDs via 1-ethyl-3-(3-dimethylaminopropyl) carbodiimide/*N*-hydroxysulfosuccinimide (EDC/Sulfo-NHS, Sigma Aldrich, USA) cross-linking. To do this, the electrode was immersed into a solution of EDC and Sulfo-NHS (1:3 wt.  %) diluted in 1 mL of phosphate-buffered saline (PBS, 0.1 M and pH 7.0) for 0.5 h in order to activate the ZnS:Mn QDs. The electrode was then transferred to a solution consisting of 3 mg Tyr enzyme and 2 mL PBS for 2 h and kept at 4 °C. The electrode was next rinsed with PBS to remove any unbound enzyme.

Electrochemical responses were analyzed by current voltammetry (CV) and conducted at room temperature using an SP-150 potentiostat/galvanostat and EC-Lab Express software purchased from BioLogic Science Instruments. A three-electrode configuration was used in this study, and the electrodes were purchased from Bioanalytical Systems Inc., USA. The fabricated Pt/SAM/QD/Tyr was used as the working electrode. Ag/AgCl 3 M KCl and Pt wire were used as the reference and auxiliary electrodes, respectively. The voltammograms were collected from −0.4 to 1.2 V with a scan rate of 50 mV s^−1^ and at concentrations of H_2_O_2_ ranging from 0 to 100 μM.

### Determination of singlet oxygen

The ^1^O_2_ generation was verified by photooxidation of 1,3-diphenylisobenzofuran (DPBF, 97 %, Sigma Aldrich, USA) and monitored by absorption. A stock solution of capped QDs at 10 mg/mL and 400 μL of 6 × 10^−5^ M DPBF (^1^O_2_ sensor) in ethanol were gradually mixed in 2 mL of HPDW and in 2 mL of buffer separately. The mixture was adequately transferred to a quartz cuvette, then continuous airflow supplied by a gas syringe was progressively added and homogenized by vigorous stirring. The absorbance of the samples was first recorded in the dark using an UV–Vis spectrophotometer (DU 800, Beckman Coulter), and then irradiated with a 532 nm laser (fluence ~101 mW/cm^2^) placed 0.4 m apart. Visible-light excitation was used to avoid the self-oxidation process of DPBF through absorption in the UV region. The absorption spectra were obtained every 2 s during approximately 16 s. Rose bengal (RB, dye content 95 %, Sigma Aldrich, USA) prepared at 1 × 10^−5^ M was used as control (Beltran-Huarac et al. 2010). All the experiments were done in triplicate.

### Imaging *P. aeruginosa* cells

In order to bind the QDs to the phosphoryl and carboxyl groups present in the cellular wall for bacterial imaging applications, the QDs were positively charged by means of protonated amine groups of chitosan (high purity, *M*_v_ 110–150 kDa, Sigma Aldrich, USA) following a similar approach described above by MPA.^51^ For batch cell cultures, the *P. aeruginosa* strain (pellets with a mean assay value of 1.0–9.9 × 10^3^ CFU purchased from MicroBiologics, USA, 0353E3) with an initial concentration of 5.4 × 10^3^ CFU/mL was diluted in 15 mL of nutrient broth and incubated at 35 °C for 48 h. The growth curves were obtained by mixing 5 mL of inoculated *P. aeruginosa*, 5 mL of QDs (40 mM), and 90 mL of nutrient broth, and then incubated at 37 °C under gentle shaking (110 rpm) for 3 h. For negative control, the QDs were replaced by 5 mL of HPDW. In order to monitor the agglomeration and PL of QDs dispersed in nutrient broth, 5 mL of QDs (40 mM) were added to 95 mL of nutrient broth. The absorbance for both the QDs incubated with or without bacteria was monitored and recorded using a UV–Visible spectrophotometer (Helios, 640 nm), until reaching the stationary phase in the cell population growth curve. No substantial difference was observed in the log and death phases. After incubation, the cell–QDs suspension was washed and precipitated for 30 min. Three mL of re-dispersed precipitate and supernatant were then collected for absorbance measurements. Further washing and gentle sonication were applied to the solution to separate the QDs attached to cellular membranes. For confocal microscopy imaging, 200 μL of each solution (bacteria–QDs, control and QDs) was added dropwise on a chamber-covered glass cell (Thermo Scientific, Nunc Lab-Tek II) and then excited at 405 nm. The confocal images were recorded using a Zeiss observer Z-1, a laser LSM 510 META using a magnification of 100×, and a U filter set, and were processed through ZEN Lite software developed by Carl Zeiss MicroImaging GmbH.

### MTS cell viability assay

The cytotoxicity effects of ZnS:Mn QDs were performed on HeLa cells using the CellTiter 96^®^ AQ_ueous_ One Solution Cell Proliferation Assay (Promega). HeLa cells were cultured in Eagle’s minimum essential medium supplemented with 10 % fetal bovine serum, 100 U/mL penicillin, 100 µg/mL streptomycin, and 250 ng/mL amphotericin B (Cellgro) at 37 °C with 5 % CO_2_. Cells were plated at a density of ca. 2 × 10^4^ in 96-well plates and grown until reaching 80–90 % confluence. Then, cell culture medium was removed and 100 µL of complete medium supplemented with QDs at different concentrations (ranging from 5.2 to 1000 µg/mL) was added, and three wells with only fresh complete medium were used as a positive control. After 24-h incubation, the medium was removed and a solution of fresh media containing 20 % of CellTiter 96^®^ AQ_ueous_ One Solution reagent was added. Wells with fresh complete medium and 3-(4,5-dimethylthiazol-2-yl)-5-(3-carboxymethoxyphenyl)-2-(4-sulfophenyl)-2H-tetrazolium (MTS) reagent without cells were used as a negative control. Afterwards, cells were incubated at 37 °C for 30 min. Then, the 96-well plates were centrifuged at 2000 rpm for 10 min. The supernatant was transferred onto a new microplate and the absorbance at 490 nm was recorded using a Synergy H1 Hybrid Multi-Mode Microplate Reader. The cell viability percentage was ascertained by means of the following equation: % = [(*A*_490_ of QD-treated cells)/(*A*_490_ of untreated cells)] × 100. All the experiments were done in triplicate.

## Results and discussion

### Characterization of the ZnS:Mn quantum dots

**Fig. 1:**
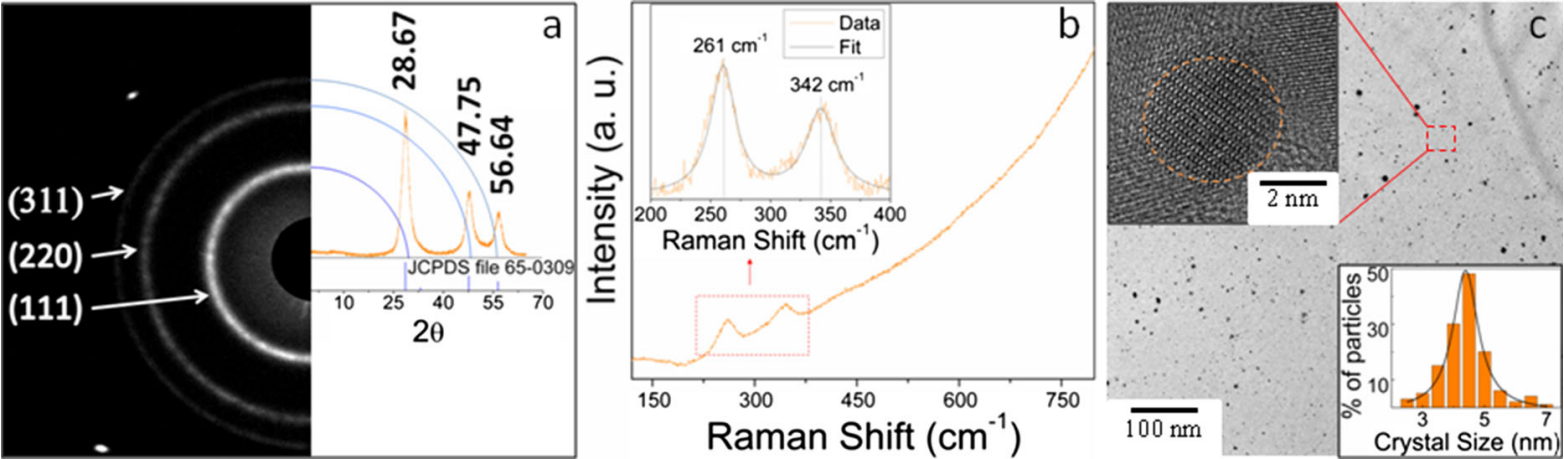
**a** XRD and SAED patterns, **b** Raman spectrum, and **c** HRTEM image of ZnS:Mn QDs. *Inset* in (**b**) shows a close-up of the Raman mode characteristic of ZnS:Mn. The *upper inset* in (**c**) shows a close-up of an individual QD. The *lower inset* in (**c**) shows the statistical size distribution of QDs

The formation and morphology of Mn-doped ZnS QDs synthesized via a one-pot wet chemical method are depicted in Fig. 1. The XRD and selected area electron diffraction (SAED) patterns show three major diffraction peaks and rings, which are indexed to the diffraction planes of zinc blende ZnS [(111), (220), and (311)] according to the JCPDS card, File No. 65-0309 and space group (see Fig. 1a). Both techniques confirmed that ZnS:Mn crystallized preferentially in the more stable cubic ZnS phase. No detectable lattice distortion (shift) was observed in the host ZnS indicative of the successful random substitution of Mn^2+^ into Zn^2+^ sites at 5 wt% Mn doping. The well-defined peaks and rings found and the absence of parasitic phases and signal of ZnS wurtzite in the XRD and SAED patterns evidence that ZnS:Mn QDs are of high crystalline quality and purity. The calculated lattice constant of ZnS:Mn was *a* = 0.5384 ± 0.0007 nm consistent with its standard value (Beltran-Huarac et al. 2013a, b). The prominent broadening of the XRD diffraction peaks is ascribed to the nanocrystalline nature of the QDs. The average crystallite size was calculated to be ~4 nm by means of Scherrer’s formula.

The microstructure quality of ZnS:Mn QDs was studied by Raman scattering spectroscopy (Fig. 1b). The well-pronounced low-frequency Raman bands observed were simulated using the damped harmonic oscillator phonon model (DHOPM) (Beltran-Huarac et al. 2014). From the simulation, the Raman modes are determined to be at 261 and 342 cm^−1^, which can be ascribed to the characteristic transverse and longitudinal optical modes of cubic ZnS, respectively (Nilsen 1969). The corresponding fit shows that those bands are red-shifted by ~10 cm^−1^ and narrow (full width at half maximum, FWHM ~23 cm^−1^) when compared to bulk ZnS (Nilsen 1969), which clearly indicates that the zinc blende ZnS is under tensile stress as a result of the growth process. The morphology and size distribution of ZnS:Mn QDs were studied by electron microscopy. The HRTEM images depicted in Fig. 1c show well-dispersed QDs with a high degree of crystallinity whose sizes range from 2 to 7 nm. A closer look of representative QDs shows that they are almost spherical with lattice fringes readily observable and a diameter of ~4 nm. The surfaces of QDs were clean, smooth, and atomically resolved with *d*-spacing of 0.31 nm corresponding to the major plane (111) of ZnS, consistent with the XRD and SAED analyses. The statistical size distribution (see lower inset of Fig. 1c) obtained by image analysis further confirmed that the average size of QDs was ~4 nm in accordance with the XRD results. Taken altogether, the XRD, Raman, and HRTEM analyses indicate the formation of narrowly distributed high-quality ZnS:Mn QDs with a zinc blende structure.

### Detection of reactive oxygen species (hydrogen peroxide)

**Fig. 2:**
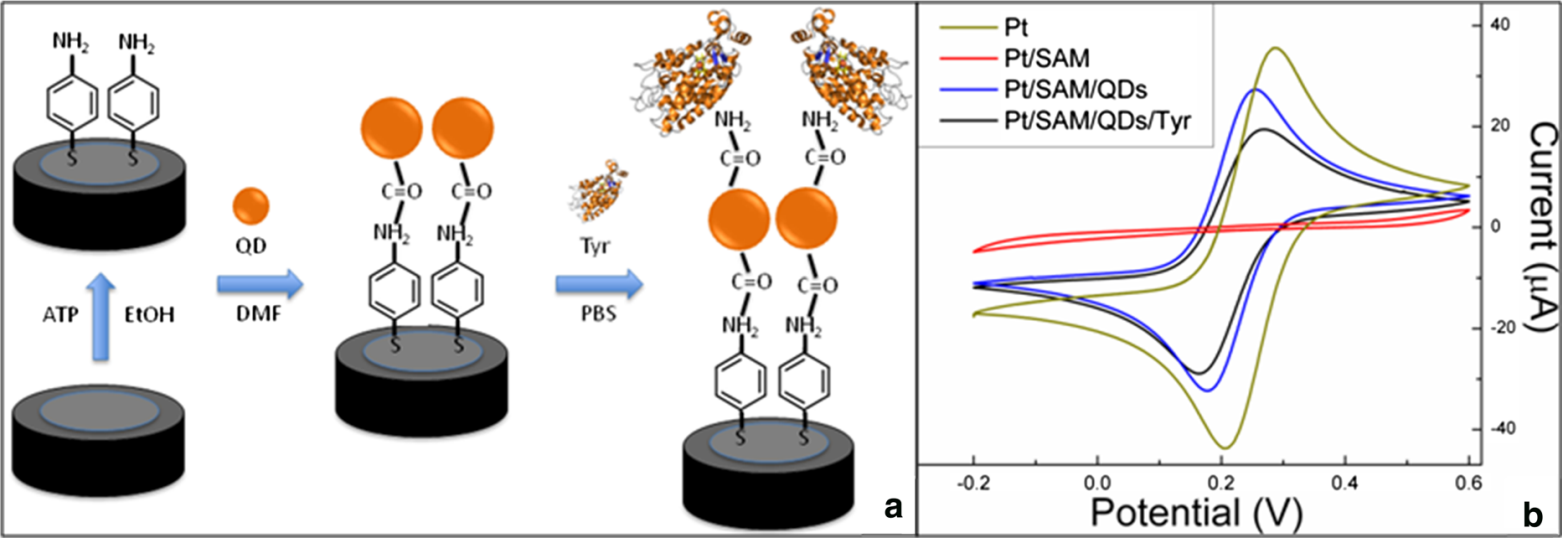
Stepwise assembly process for the biosensor fabrication. **a** Main steps of the immobilization of Tyr onto ZnS:Mn QDs and **b** their corresponding cyclic voltammograms for the electrochemical detection of H_2_O_2_ in a 3 M KCl containing 10 mM ferricyanide at a scan rate of 50 mV s^−1^

The assembly of the amperometric biosensor based on Tyr enzyme immobilized onto ZnS:Mn QDs to detect H_2_O_2_ is depicted in Fig. 2. Prior to the covalent immobilization, the surface of the as-cleaned polycrystalline Pt electrodes was modified using 4-ATP that enables the formation of a NH_2_-terminated AM, followed by covalent linkage of QDs. The carboxylic acid groups on the surface of QDs were crosslinked to the primary amines of enzyme through an EDC-mediated coupling, allowing for a strong covalent immobilization of Tyr onto QDs (see Fig. 2a). Each immobilization step was monitored by CV via direct detection of the electron transfer of the active ferrocyanide species present in the solution, as shown in Fig. 2b. The voltammogram of the bare Pt electrode shows an anodic peak at 280 mV and a cathodic peak at 220 mV that arises from [Fe(CN)_6_]^4−^ and [Fe(CN)_6_]^3−^, respectively, and is characteristic of the quasi-reversible one-electron transfer redox behavior of the electroactive species. It was observed that such redox peaks disappear when the 4-ATP electrode modification occurs, which is attributable to the insulating effect produced by the densely packed SAM formation on the electrode surface that hinders the interfacial charge transfer between the ferricyanide ion and the electrode. SAM formation provides the chemical environment necessary to covalently attach MPA-capped QDs onto the electrode. A relative increase in current was detected in Pt/SAM/QDs, which was associated to the electron injection into sub-bandgap states of the metal (Zn,Mn) sulfide (Topoglidis et al. 2001; Boschloo and Fitzmaurice 1999). A decrease in current was observed when Tyr (IEP of ~4.7–5.0) is covalently linked to the QDs. This is due to the enzymatic blockage that is produced on the surface of QDs, thus corroborating both the enzyme deposition and the proposed biosensor assembly (Hernandez-cancel et al. 2015) (see Fig. 2a).

**Fig. 3:**
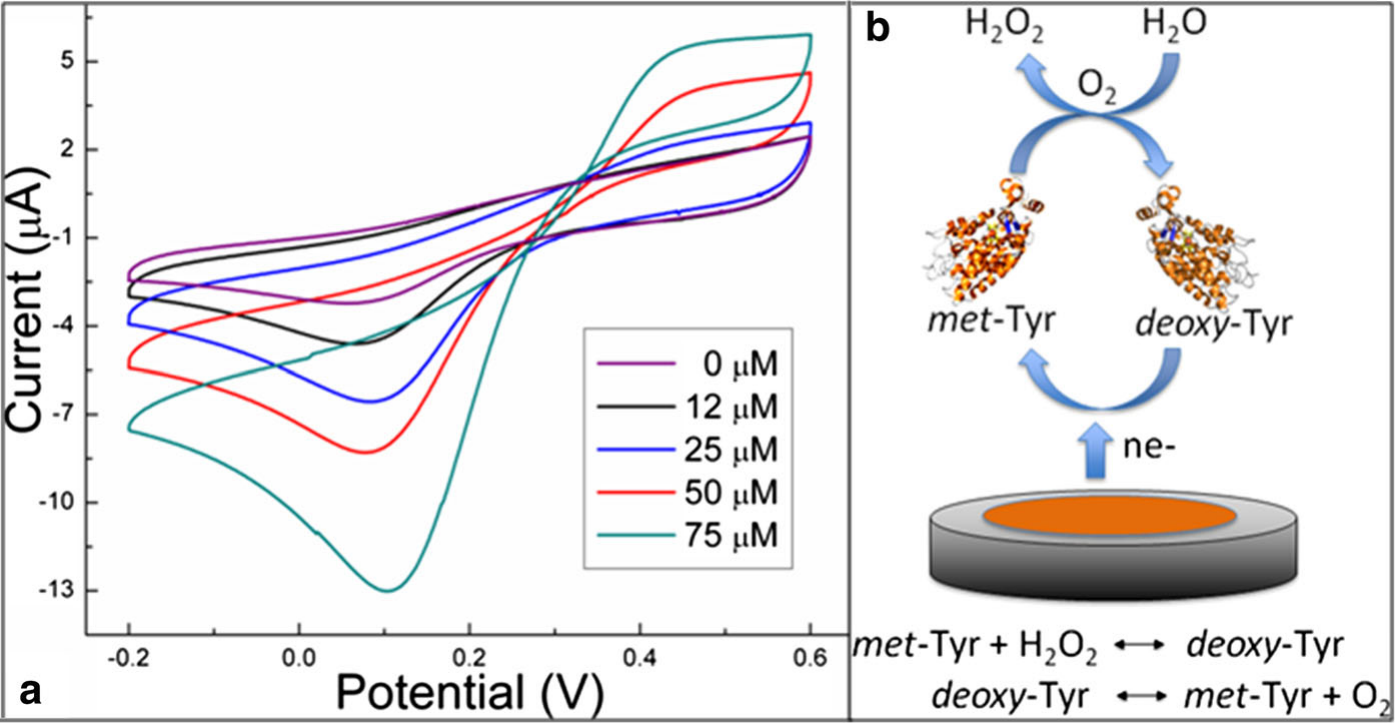
**a** H_2_O_2_–Tyr biosensor in the presence of 0.1 M PBS at pH 7.0 and a scan rate of 50 mV s^−1^. **b** Catalase-cycled kinetic mechanism for H_2_O_2_ biosensor based on ZnS:Mn/Tyr

Figure 3a shows the electrocatalytic response of the biosensor to H_2_O_2_ at concentrations ranging from 0 to 75 μM and in the presence of 0.1 M PBS. An enzyme redox peak centered at ~63 mV in the absence of H_2_O_2_ was observed, which signifies that the electroactive prosthetic group of the enzyme is present in close proximity to the modified electrode. An enhancement of the anodic peak current (~4.61 μA) was obtained when a 12 μM H_2_O_2_ solution was gradually added, indicating that the current response of the biosensor represents the characteristic electrochemical behavior of H_2_O_2_ (Kafi et al. 2008), as expected. This is due mainly to the catalytic effect of Tyr in the absence of a one-electron donor substrate, which remains active and exhibits an enhanced stability as a result of the effective immobilization of ZnS:Mn QDs. At higher concentrations (as high as 75 μM), a net anodic current of ~13.01 μA was detected. This demonstrates that ZnS:Mn-immobilized Tyr exhibits improved catalytic properties in the reduction of H_2_O_2_, and is partially ascribed to the increase of binding sites of the QDs for enzyme loading. It is noteworthy that although the catalytic cycle of Tyr involves H_2_O_2_ consumption and water generation (see Fig. 3b), no inactivation of Tyr was observed up to 100 μM of H_2_O_2_ (a current of ~18.18 μA). This tendency clearly indicates that H_2_O_2_ is acting as both oxidizing and reducing substrate in solution and is not irreversibly adsorbed on the biosensor surface, i.e., the reaction between H_2_O_2_ and Tyr is a diffusion-controlled process at this scan rate. The catalase activity found in mushroom Tyr/QDs for H_2_O_2_ can be understood in terms of a catalase-cycled kinetic mechanism, as follows. It is known that under anaerobic conditions Tyr or polyphenol oxidase exhibits two enzymatic species, *met*-Tyr and *deoxy*-Tyr, which can initiate the reaction indistinctly. Thus, a H_2_O_2_ molecule of the solution at pH 7.0 can reduce *met*-Tyr to *deoxy*-Tyr through the formation of *oxy*-Tyr, which releases oxygen into the medium and is subsequently transformed into *deoxy*-Tyr. Then, this is oxidized to *met*-Tyr upon binding another H_2_O_2_ molecule (see Fig. 3b). In this context, H_2_O_2_ can be bound to the axial position of one of the active copper atoms of the *oxy*-Tyr site during the catalase cycle to produce *deoxy*-Tyr, which correlates well with the recent results obtained for biosensors based on Tyr/ZnO (Rather et al. 2014; Garcia-Molina et al. 2005). This is also consistent with the preparation of binuclear copper active sites of tyrosinase, being able to act on both monophenols and *o*-diphenols in the oxygenated form, *oxy*-Tyr, whereas the *met* and *deoxy* forms need the assistance of oxygen and H_2_O_2_ (Garcia-Molina et al. 2005).

### Generation of reactive oxygen species (singlet oxygen)

**Fig. 4:**
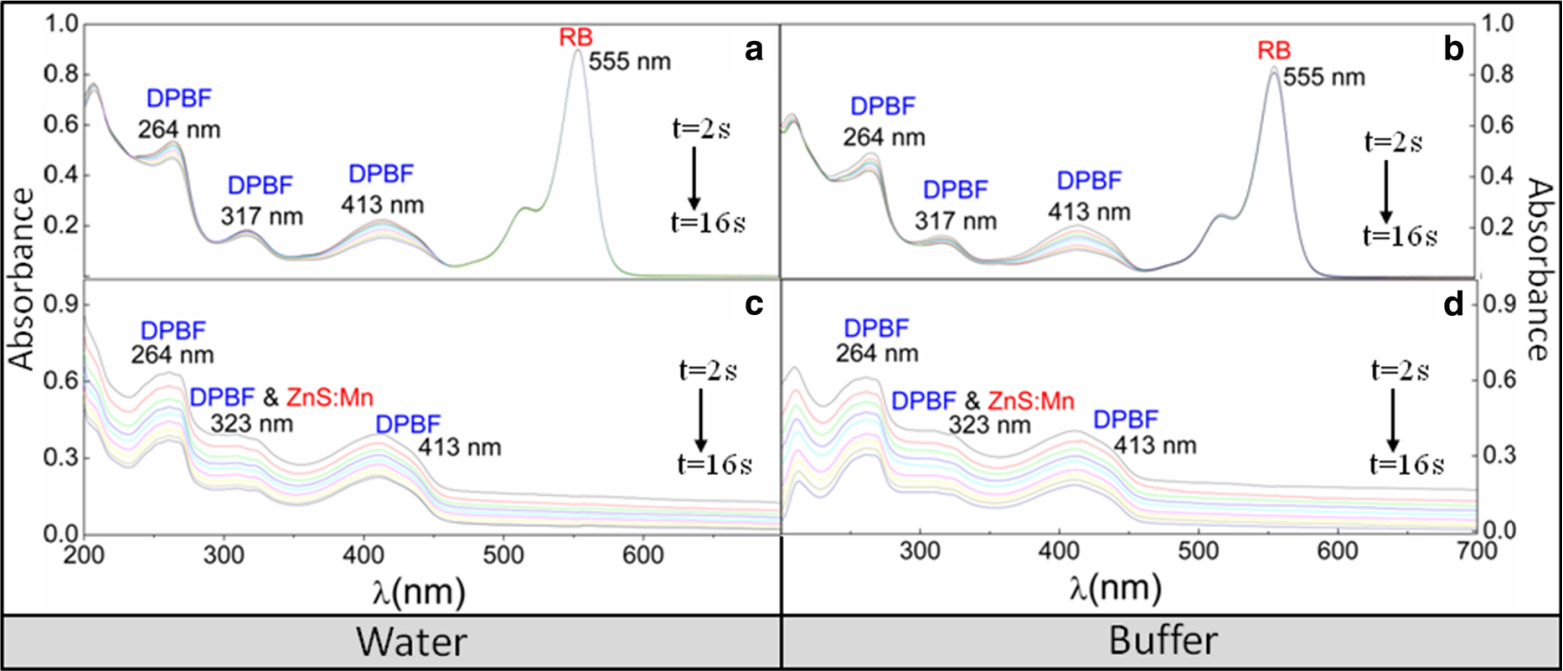
Absorption spectra of the DPBF oxidation (at 6.0 × 10^−5^ M) in the presence of RB (at 1.0 × 10^−5^ M) and ZnS:Mn QDs (10 mg/10 mL) in air-saturated **a**, **c** water and **b**, **d** buffer solutions under vigorous stirring

In the previous discussion, we furnished evidence on the possibility of detecting ROS, such as H_2_O_2_, based on Tyr and ZnS:Mn QDs. We evaluate here the ability of QDs to generate ROS when used as PS, employing both a chemical scavenger (DPBF, a sensor highly sensitive to ^1^O_2_) and a standard PS (RB, a dye with a known ^1^O_2_ quantum yield) in organic and aqueous media and through a photooxidation approach. To do this, we specifically chose the RB/DPBF pair system because its photo-oxygenation pathway can solely produce ^1^O_2_ via a Type-II mechanism (Beltran-Huarac et al. 2010; Ding et al. 2011). Thus, using ZnS:Mn as a PS, excited singlet-state oxygen can be produced by energy transfer, which in turn oxidizes the ground-state substrate. Figure 4 depicts the absorption spectra of the DPBF oxidation photosensitized by RB and ZnS:Mn QDs in water and buffer solution, when exposed to 532 nm laser irradiation for every 2 s under vigorous stirring. In order to confirm that the reaction is induced by ^1^O_2_ instead of a direct interaction between QDs and DPBF, air-saturated solutions were prepared. Oxygen-depleted solutions (via inert gas purge) showed no detectable changes in the absorption spectra. Figure 4a depicts the absorption profile of DPBF (400 μL of a 6.0 × 10^−5^ M solution) in 2 mL of water in the presence of RB (1.0 × 10^−5^ M). This spectrum shows the typical absorption band edges of DPBF centered at 264, 317, and 413 nm, and of RB peaking at 555 nm (Beltran-Huarac et al. 2010, 2013a, b; Tuncel et al. 2011). No photooxidation of RB was observed when the samples were exposed to light (10 mW) for 16 s, as expected. However, the absorption bands of DPBF dropped significantly indicating that the scavenger is being oxidized by singlet oxygen. A more conspicuous photooxidation was seen in the broader absorption band centered at 413 nm, which is used as a reference for the ^1^O_2_ QY calculation. A similar behavior but with faster reaction rates was observed when a buffer solution is used instead of water (see Fig. 4b). Figure 4c depicts the absorption profile of DPBF (400 μL of a 6.0 × 10^−5^ M solution) in 2 mL of water in the presence of ZnS:Mn QDs (10 mg/10 mL). This spectrum shows the typical absorption band edges of DPBF centered at 264 and 413 nm, and of ZnS:Mn peaking at 323 nm (Beltran-Huarac et al. 2010, 2013a, b). It was difficult to differentiate the absorption band of DPBF at 317 nm given that the spectral linewidth of the ZnS:Mn absorption band was further increased. A more regular DPBF oxidation with larger changes of absorption was also observed, when compared to RB spectra, suggesting that the photoluminescent QDs are multiplet-state photoactive and can be used as a stable, more efficient PS. A similar behavior but with faster reaction rates was observed when the buffer solution is used instead of water (see Fig. 4d). Note that the dependence of the reaction on dissolved O_2_ indicates that ^1^O_2_ is generated by the QDs during the photosensitization. No oxidation of DPBF was detected in the absence of light.

**Fig. 5:**
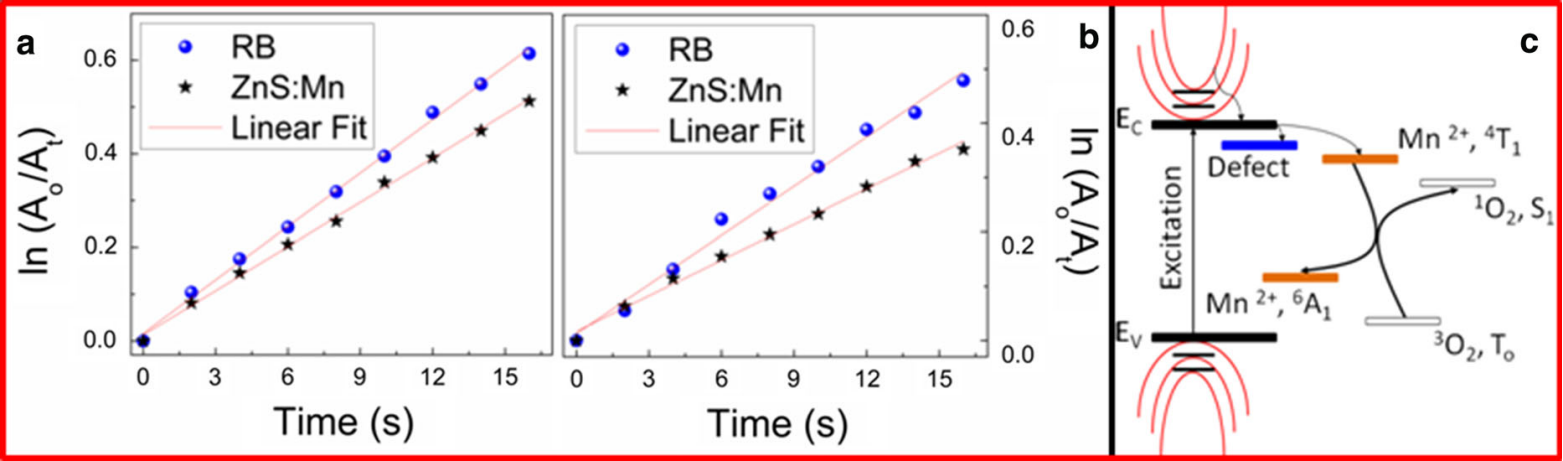
Decay curves of the 413 nm absorption band of DPBF (6.0 × 10^−5^ M) induced by singlet oxygen, which are produced by RB and ZnS:Mn QDs at 15 μg/mL in air-saturated **a** buffer and **b** water. **c** Schematic of the chemical trapping mechanism proposed for the generation of singlet oxygen

In order to calculate the ^1^O_2_ QY (*Φ*_Δ_) of the photoexcited ZnS:Mn QDs in organic and aqueous media, we have expressed the oxidation of DPBF in terms of the decrease of absorbance (*A*) at 413 nm as a function of time (Δ(ln[*A*_o_*/A*_*t*_]/Δ(*t*)). The profiles of the time-dependent oxidation of DPBF induced by singlet oxygen, obtained by this procedure, are depicted in Fig. 5a, b. It was observed that ZnS:Mn shows high reaction rates and a marked linear correlation, i.e., a first-order kinetic reaction, which is a clear indicator that ZnS:Mn QDs can yield high ^1^O_2_ values in both buffer and water. To quantify this observation, we employ a widely validated chemical trapping method (Beltran-Huarac et al. 2010; Nardello et al. 1997; Xiao et al. 2011; Nadhman et al. 2014) that consists in the comparative use of a standard PS of a known *Φ*_Δ_ (*Φ*_Δstandard_), such as RB, and the slopes determined from the decay curves of a chemical scavenger of ^1^O_2_, such as DPBF, which irreversibly undergoes a 1,4-cycloaddition that is detected as an intensity drop of its absorption band at 413 nm (Xiao et al. 2011). Thus, the *Φ*_Δ_ (number of ^1^O_2_ molecules formed per absorbed photon) of ZnS:Mn QDs (*Φ*_ΔZnS:Mn_) is calculated, as follows (Nardello et al. 1997):

1 $$\varPhi_{{\Updelta {\text{ZnS:Mn}}}} = \left( {\varPhi_{{\Updelta {\text{standard}}}} \cdot k_{{\Updelta {\text{ZnS:Mn}}}} } \right)/k_{\text{standard}} ,$$

where *k*_standard_ and *k*_ZnS:Mn_ represent the slopes determined from the decay curves for RB and ZnS:Mn QDs, respectively. By introducing the value of *Φ*_Δstandard_ (RB = 0.76) (Xiao et al. 2011) and the determined slopes, one obtains *Φ*_ΔZnS:Mn_ = 0.62 ± 0.02 in buffer and *Φ*_ΔZnS:Mn_ = 0.54 ± 0.03 in water. These values are higher than those reported for some semiconductors and metals employed as PSs (see Table 1). Thus, based on our observations, we suspect that the ^1^O_2_ generation by ZnS:Mn QDs in water and buffer solution involves the following (Beltran-Huarac et al. 2013; Beltran-Huarac et al. 2010; Xiao et al. 2011; Nadhman et al. 2014; DeRosa and Crutchley 2002): (i) the promotion of electrons toward the conduction band of ZnS:Mn by light excitation; (ii) nonradiative decay of the photoexcited QDs into a multiplet energy state, ^4^*T*_1_ (first excited state with spin 3/2), due to the internal Mn^2+^ ion transition; (iii) intersystem crossing of QDs to a long-lived multiplet state, ^6^*A*_1_ (ground state with spin 5/2); (iv) energy transfer from the multiplet state of QDs to the triplet ground state (*T*_o_) of oxygen (^3^O_2_); and (v) subsequent yield of singlet oxygen (^1^O_2_), which irreversibly reacts with DPBF via a 1,4-cycloaddition yielding an endoperoxide that decomposes in turn to produce o-dibenzoylbenzene. A schematic of these processes is depicted in Fig. 5c. Given that the reaction occurs in solution, we assume that the excited state (^1^O_2_) eventually relaxes back to the ground state (^3^O_2_) via nonradiative deactivation (Hurst and Schuster 1983). In this proposed mechanism, no response of hydroxyl radical and superoxide species was considered since the DPBF is highly selective for ^1^O_2_. We believe that the powerful visible light supplied by the laser which is used to avoid the self-oxidation process of DPBF (through absorption) and assisted by the 150 mW continuous ozone-free Xe lamp light (200–800 nm) can induce energy transfer from the particles to the molecular oxygen and DPBF. Taken together, this method provides an efficient pathway to generate ^1^O_2_, which rapidly reacts with unsaturated carbon–carbon bonds present in DPBF via the energy transfer from the ^4^*T*_1_ state to molecular oxygen: ^4^*T*_1_ (PS) + ^3^O_2_ → ^6^*A*_1_ (PS) + ^1^O_2_, wherein ZnS:Mn QDs act as an efficient PS.

**Table 1:** Production of ^1^O_2_ *Φ*_Δ_ for some semiconductors and metals used as PSs

Semiconductor/metal	^1^O_2_ *Φ*_Δ_	Reference
CdSe	0.05	Samia et al. (2003)
Si NPs*	0.11	Xiao et al. (2011)
ZnO:Ag	0.28	Nadhman et al. (2014)
Fe_3_O_4_/ZnO	0.28	Beltran-Huarac et al. (2010)
CdSe/CdS/ZnS/dye	0.31	Tsay et al. (2007)
CdTe/dye	0.43	Shi et al. (2006)
ZnS:Mn	0.62 ± 0.02	Present work

* Porous nanoparticles

### Cytotoxicity and cell imaging

**Fig. 6:**
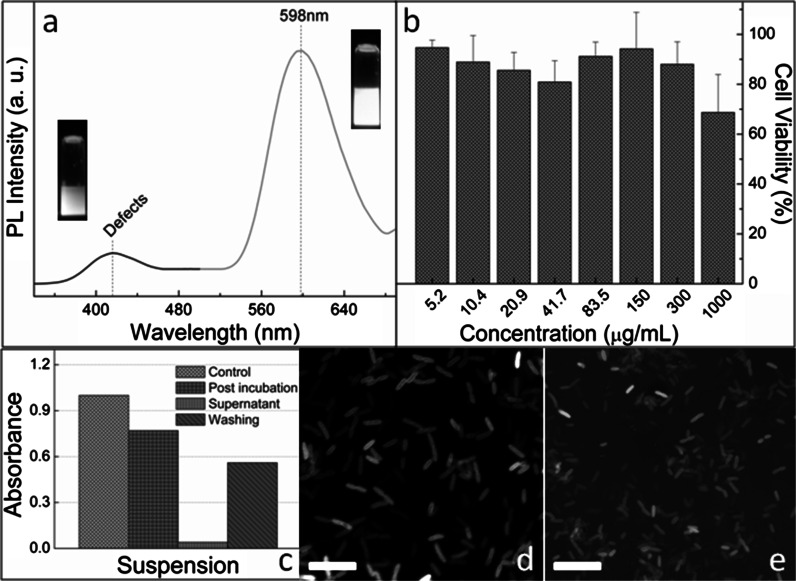
**a** PL spectrum of an aqueous solution of ZnS:Mn QDs. The *insets* show the optical images of ZnS (*blue*) and ZnS:Mn (*orange*) QDs dispersed in water as viewed under UV light. **b** HeLa cell viability in the presence of ZnS:Mn QDs at different concentrations after incubation for 24 h. **c** Absorbance at 640 nm of *P. aeruginosa* cells incubated with 5 mL of an ZnS:Mn solution at 40 mM. **d** Representative bright-field image of unlabeled *P. aeruginosa* cells and **e** confocal microscopy image of ZnS:Mn-labeled *P. aeruginosa* cells. *Scale bars* 5 μm. (Color figure online)

In the previous discussion, we have accounted for the high ^1^O_2_ QY of ZnS:Mn QDs in terms of a chemical trapping approach, which in turn involves a high PL QY that promotes the ^4^*T*_1_–^6^*A*_1_ transition. The PL QY of ZnS:Mn QDs falls in the range of 13.2–70 % at room temperature with a decay time of a few milliseconds (Shen et al. 2012; Beltran-Huarac et al. 2013a, b; Geszke-Moritz et al. 2011, 2013; Bhargava and Gallaguer 1994). In this section, we further exploit additional PL characteristics of ZnS:Mn and evaluate their cytotoxicity in order to employ them as luminescent nano-probes for imaging *P. aeruginosa* cells. The PL spectrum of ZnS:Mn QDs dispersed in water is depicted in Fig. 6a, which shows two emission bands in the visible region. The blue band at ~416 nm (labeled as defects) is ascribed to ZnS host, corresponding to the self-activated recombination centers arising from shallow donors and zinc vacancies (Beltran-Huarac et al. 2013a, b), consistent with the photooxidation analysis. The orange emission band at ~598 nm is due to the internal Mn^2+^ ion transition, which causes energy transfer from the *s*-*p* electron–hole pair band states (ZnS host) to the Mn^2+^ ion *d*-electron states (Beltran-Huarac et al. 2013a, b). Both bands in the PL spectrum confirm the homogeneous incorporation of Mn^2+^ ions into the semiconductor. The optical images of ZnS QDs (blue) and ZnS:Mn QDs (orange) in aqueous solution when exposed to UV light are shown in the inset of Fig. 6a. Prior to evaluating ZnS:Mn QDs as luminescent nano-probes for bioimaging, we first study their cytotoxic effect on human cervical adenocarcinoma (HeLa) cells. Cell viability was examined through the measurement of cell metabolic activity. The mitochondrial function was measured via MTS viability assay after incubating HeLa cells with different concentrations (5.2–1000 µg/mL) of QDs for 24 h. Viable cells convert MTS tetrazolium into formazan dyes, which can be spectrophotometrically detected (Cory et al. 1991). The cell viability results are depicted in Fig. 6b. It was observed an average of 95 % of viable cells at 5.2 µg/mL of QDs, and 88 % at 300 µg/mL. The cell viability slightly decreased until a value of ~68 % at an excessively high concentration (1000 µg/mL). These findings reflect that ZnS:Mn QDs are not toxic for human cells and cause a negligible cytotoxicity (~32 % of reduction in cell viability) at elevated doses, which are not expected to be administered in clinical trials. The analysis of variance (ANOVA) test indicated that there are no statistically significant differences attributed to QDs’ concentrations (*p* = 0.1457). To evaluate the ability of ZnS:Mn QDs as luminescent nano-probes, we incubated *P. aeruginosa* cells with 5 mL of QD solution at 40 mM for 3 h, and monitored both the corresponding suspensions by measuring the optical absorbance and the internalization of the QDs by the cells via confocal microscopy. To do this, we made the QDs positively charged by protonating the amine groups of chitosan, which bind to the phosphoryl and carboxyl groups present in the cellular walls. Figure 6c shows the suspensions of cells (control) and chitosan-capped QDs–cells monitored by absorption at 640 nm. The decrease in the absorbance of the post incubation process indicates that the QDs were able to either attach to the cells or move inside the cells from the suspension. After washing, the supernatant showed a slight absorbance, as expected, whereas the remaining suspension showed an absorbance of ~55 %, indicating that QDs were able to enter the cells. The internalization of the 4 nm ZnS:Mn QDs by the cells was confirmed by confocal microscopy, as shown in Fig. 6d, e. It was clearly observed that the QD-labeled cells emit a stronger fluorescent signal (reflected as high-contrast images) when compared to the control (unlabeled cells), revealing the dimension and rod-like morphology characteristic of *P. aeruginosa* strains, which assume a wide range of morphologies and lengths dependent on the growth parameters. The well-defined geometry of the cells suggests that the fluorescence signature was homogeneously distributed throughout the bacterial cell during incubation. No photobleaching effect was observed after a few hours of continuous irradiation exposure, evidencing that the QDs are an ideal alternative to conventional organic dyes that are highly susceptible to light-induced oxidation. Even though the amine groups present in the QDs serve as effective sites for bioconjugation, further studies are needed to fully understand the nature of the molecular associations of the chitosan-capped ZnS:Mn QDs with the *P. aeruginosa* cells.

## Conclusion

In summary, we have synthesized ZnS:Mn QDs in aqueous solution at room temperature for multiple types of biological detection and enzyme immobilization. Our findings indicate that ZnS:Mn-immobilized Tyr biosensor is able to detect not only phenols but also hydrogen peroxide at concentrations below 12 μM. A correlated synergistic effect was observed between the high catalytic activity of Tyr and the large surface area of QDs, which resulted in an enhanced electrochemical response. Through an indirect assay to monitor the singlet oxygen quantum yield, it was evidenced that ZnS:Mn QDs can be used as an efficient photosensitizer for photodynamic therapy exhibiting a ^1^O_2_ QY of 0.62 ± 0.02 in buffer and 0.54 ± 0.03 in water. HeLa cells being exposed to ZnS:Mn QDs for 24 h show high tolerance reaching cell viabilities as high as 88 % at 300 µg/mL. The ability of chitosan-capped ZnS:Mn QDs to penetrate microbial cells and serve as luminescent nano-probes makes them suitable to be used as phylogenetic oligonucleotide probes for single-cell detection. Our observations furnish evidence on the possibility to avoid the use of organic acid-stabilized Cd-based systems and substitute their optical contribution with Mn doping in the ZnS system for bioimaging, offering a promising biomaterial for expanding the biomedical applications of semiconducting nanocrystals, and bringing forth new arenas to manipulate their theranostic capabilities.
